# Tuberculosis contact investigation with a new, specific blood test in a low-incidence population containing a high proportion of BCG-vaccinated persons

**DOI:** 10.1186/1465-9921-7-77

**Published:** 2006-05-17

**Authors:** R Diel, A Nienhaus, C Lange, K Meywald-Walter, M Forßbohm, T Schaberg

**Affiliations:** 1 School of Public Health, University of Düsseldorf, Germany; 2 Institution for statutory accident insurance and prevention in the health and welfare services, Hamburg, Germany; 3 Research Center Borstel, Division of Clinical Infectious Diseases, Germany; 4 Public Health Department Hamburg-Mitte, Germany; 5 Public Health Department Wiesbaden, Germany; 6 Center of Pneumology, Deaconess Hospital Rotenburg/Wümme, Germany

## Abstract

**Background:**

BCG-vaccination can confound tuberculin skin test (TST) reactions in the diagnosis of latent tuberculosis infection.

**Methods:**

We compared the TST with a *Mycobacterium tuberculosis *specific whole blood interferon-gamma assay (QuantiFERON^®^-TB-Gold In Tube; QFT-G) during ongoing investigations among close contacts of sputum smear positive source cases in Hamburg, Germany.

**Results:**

During a 6-month period, 309 contacts (mean age 28.5 ± 10.5 years) from a total of 15 source cases underwent both TST and QFT-G testing. Of those, 157 (50.8%) had received BCG vaccination and 84 (27.2%) had migrated to Germany from a total of 25 different high prevalence countries (i.e. >20 cases/100,000). For the TST, the positive response rate was 44.3% (137/309), whilst only 31 (10%) showed a positive QFT-G result. The overall agreement between the TST and the QFT-G was low (κ = 0.2, with 95% CI 0.14.-0.23), and positive TST reactions were closely associated with prior BCG vaccination (OR 24.7; 95% CI 11.7–52.5). In contrast, there was good agreement between TST and QFT-G in non-vaccinated persons (κ = 0.58, with 95% CI 0.4–0.68), increasing to 0.68 (95% CI 0.46–0.81), if a 10-mm cut off for the TST was used instead of the standard 5 mm recommended in Germany.

**Conclusion:**

The QFT-G assay was unaffected by BCG vaccination status, unlike the TST. In close contacts who were BCG-vaccinated, the QFT-G assay appeared to be a more specific indicator of latent tuberculosis infection than the TST, and similarly sensitive in unvaccinated contacts. In BCG-vaccinated close contacts, measurement of IFN-gamma responses of lymphocytes stimulated with *M. tuberculosis*-specific antigen should be recommended as a basis for the decision on whether to perform subsequent chest X-ray examinations or to start treatment for latent tuberculosis infection.

## Background

Even in countries with a low tuberculosis (TB) incidence, population-based studies have recently revealed a high frequency of transmission of *Mycobacterium tuberculosis *(MTB) by applying classical epidemiological and molecular strain-typing techniques [1-5]. Routine contact tracing of infectious TB cases to detect individuals at high risk of being exposed to MTB, and to offer treatment for them if they test positive for latent TB infection (LTBI), are key components of TB control programs in developed countries. Although the epidemiological logic of treating LTBI – aiming to decrease the incidence of TB and subsequently diminish further MTB transmission – is clear, the success of treating populations at TB risk has been limited by the lack of a definitive diagnostic test for LTBI.

The tuberculin skin test (TST) introduced by Mantoux has been widely used as the screening test of choice to identify individuals with LTBI for more than a century. One of its intrinsic problems, however, is its cross-reactivity with antigens present in other mycobacteria, such as the *Mycobacterium bovis *bacillus Calmette-Guerin (BCG) vaccine strain, the most widely used vaccine ever, and non-tuberculous mycobacterial (NTM) species. Therefore, alternative diagnostic tools for the detection of LTBI have been explored. Two proteins from *M. tuberculosis*, ESAT-6 and CFP-10, stand out as suitable antigens that induce a strong IFN-γ-secreting CD4 T-cell-mediated immune response to infection [6,7] and are absent in *M. bovis *BCG and most NTM's, with the exceptions of *M. szulgai*, *M. marinum*, and *M. kansasii*.

A new diagnostic test for LTBI, QuantiFERON^®^-TB Gold In Tube (QFT-G), employs whole blood collected and incubated with overlapping peptides representing the TB antigens ESAT-6 and CFP-10 along with a peptide from another TB-specific antigen, TB7.7 (Rv 2654). For this version of QFT-G, evacuated tubes are pre-coated with control and test antigens and the blood-collection tubes also serve as the incubation vessels. The QFT-G test measures the amount of IFN-γ produced by Tcells previously exposed to MTB when they are stimulated with the TB-specific antigen during overnight incubation. Whereas the sensitivity of the QFT-G assay appears at least comparable to that of the TST for the detection of active MTB disease [8,9], the specificity of the test has been demonstrated as superior to that of the classical TST, especially in BCG-vaccinated persons [8,10]. However, the efficacy of the QFT-G test for detecting LTBI in recent contacts of infectious source cases has so far only been addressed in a country with intermediate MTB incidence [10] (as opposed to the low MTB incidence encountered in most European countries and the USA) or in a predominantly teenage group [11], or in studies involving the analysis of singular outbreaks [12,13]. Until now, the QFT-G assay has not been evaluated for routine use in close contacts in a low-incidence setting.

The city of Hamburg (one of the German federal states and, with 1.73 million residents, the second largest city in Germany) is particularly affected: in 2004, Hamburg had a TB incidence rate of 12.0 per 100,000. This rate is higher than in any of the other fifteen German federal states, and in recent years, against the national downward trend, it has been rising [14]. Hamburg has also the highest proportion of foreign residents, 14.1%, compared with a German national average of 8.8% reported for 31 December 2004 [15]. By conducting an ongoing comparison study using both the Mantoux and QFT-G test simultaneously in routine contact tracing, two main questions were addressed: (i) How well do TST and QFT-G results correlate and what are the presumed reasons for divergent results? (ii) What are the consequences of actions to be taken on the basis of these results under the "real life" conditions of contact tracing in a metropolis?

## Methods

### Study population

Close contacts of sputum-smear-positive source cases were prospectively enrolled into the study over a 6-month period from May 1st to October 31st 2005. "Close" contacts were defined as household and intimate contacts; these also comprised employees who had demonstrable continuous exposure to the source case, or pupils sharing the same classroom. According to the definition of Behr *et al*. [16] in any case the total (aggregate) exposure time was not less than 40 hours prior to the diagnosis of the respective index case; the estimated minimum time of contact was recorded for each contact person in four-hour windows (40–43 hours, 44–47 hours etc.). Contacts with only occasional exposure and an exposure time less than 40 hours to the source case during the presumed period of infectiousness were not included in the study. All individuals agreed to participate in the study by written consent.

Each individual was interviewed by trained public-health staff using a standardized questionnaire. Information was obtained on: the contact's sex, date and country of birth, nationality, immigration status (if applicable), number of weeks of residence in Hamburg (if necessary, augmented by official records of the local residents' registry), current address (or whether the contact was homeless), the nature of the contact's current employment (if any), the nature of contact to the source case, the time interval between the most recent suspected exposure date and the date of diagnosis of the source case, details of any previous contact tracing, results of previous tuberculin skin testing and chest radiographic findings, BCG vaccination status (if details were unclear, this was augmented by inspection of the BCG vaccination scar), and associated medical problems (especially HIV infection).

### IFN-γ release assay and Mantoux TST

The QFT-Gin-tube methodology (Cellestis Ltd, Carnegie, Australia) involves two processes: (1)collection of whole blood into an evacuated tube containing TB-specific antigens and a control tube, and incubating overnight, and (2) measurement of IFN-γ production by ELISA in harvested plasma. Venous bloodwas collected from each subject before administration of Mantoux TST intotwo heparinised blood tubes calibrated to draw 1 ml of blood. The control tube contained onlyheparin, asa negative control; the heparinised TB antigentubecontained dried overlapping peptides representing the entire sequences of ESAT-6 and CFP-10 and a peptide from the TB antigenTB7.7 (Rv 2654, amino acids 38–55). The tubes were shaken and immediately incubated at 37°C for16–24 hours, after which they were centrifuged and plasma harvested. Plasma was kept refrigerated at 4–6°C until the ELISA was performed. The IFN-γ ELISA was performed using the method recommended by the manufacturer [17]. IFN-γ values (IU/ml) for the TB-specific antigen-stimulated plasmaswere corrected for background by subtracting the valueof the subject's respectivenegative control. The cut off value for a positive test was IFN-γ ≥0.35 IU/ml, as recommended by the manufacturer, andalso derived in previous studies [8,13].

For the TST, 0.1 ml of tuberculin 10 GT Behring (Chiron Bering, Marburg, Germany; equivalent to about 5 TU of PPD-S) was injected intradermally into the volar aspect of the forearm and transverse induration diameter was measured 72 hours later. Individuals performing the TST were blinded to QFT-G results and *vice-versa*.

### Statistical analysis

Categorical data were compared by the χ-squared test (or Fisher's exact test, when expected cell sizes were smaller than five). The Wilcoxon rank sum test was performed to determine whether the distribution of continuous variables differed between two groups. Concordance between the results of the TST and QFT-G tests was assessed by using κ coefficients, both for contacts with and without BCG vaccination. Kappa values below 0.4 indicate weak correlation, values of 0.41–0.60 indicate good agreement and values above 0.6 strong agreement [18]. Logistical regression was used to estimate odds ratios (ORs) of positive responses to each test for each of the variables measured. Variables included were age, sex, origin at birth (German or foreign), history of BCG vaccination and exposure time of the contacts to a source case. All *p *values reported are based on two-tailed comparisons, with statistical significance set at *p *< 0.05.

## Results

The contact investigations yielded a total of 311 persons with identified risk of LTBI due to their exposure to sputum-smear-positive source cases. Two contacts did not come back for their TST to be read and were not used in the final analysis. Thus, 309 contacts of a total of 15 different sputum-smear-positive source cases formed the study population, the demographic and clinical features of which are described in Table 1. None of the contacts reported that they were seropositive for HIV, undergoing haemodialysis, currently being treated with corticosteroids or other immunosuppressives, known to have a malignant disease or diabetes mellitus or having recently been immunized with live vaccines. As there was no evidence of suspicion of immunosuppression for any of the contacts, an optional PHA mitogen control tube for the QFT-G test was not performed for this study.

**Table 1:** Demographic and behavioral characteristics of the study participants

**Variables**	**n**
Age of contacts (years) – mean (± SD)	28.5 (± 10.5)
Previous BCG vaccination	157 (50.8%)
Origin (Foreign/German)	84 (27.1%)/ 225 (72.9%)
Residence time (weeks) – mean (± SD)	535.4 (± 394)
Exposure time (hours) – mean (± SD)	221 (± 273)
Previous contact tracing; TST results	8; 1 TST-positive
No. of contacts per source – mean (± SD)	20 (± 13)
Foreign places of birth	25

The number of close contacts per source case varied between 1 and 39 individuals; the mean exposure time (± SD) was 221 (± 273) hours, with a range from 40 to 1004 hours (the latter corresponding to 42 days).

Of the 309 contacts, 225 (72.8%) were born in Germany, while 84 (27.2%) had migrated to Germany from a total of 25 different countries. All of the migrants came from high-incidence MTB countries (defined as those having an incidence of 20 or more cases per 100,000 inhabitants [19]), including 20 individuals from countries of the former Soviet Union and 18 from Turkey. The mean period between the date of entry to Germany and the date of contact tracing was 535.4 ± 394 weeks, with a range from 39 to 1601 weeks. The mean age of the contacts was 28.5 ± 10.5 years (range 14–53) and there were only slightly more female contacts (*n *= 160, 51.8%) than male. Only 8 contacts had previously been involved in contact tracing or in an employment-related investigation, and of these only one had given a positive TST result at that time (and was TST-positive as well as QFT-G -positive in the present study).

**Figure 1:**
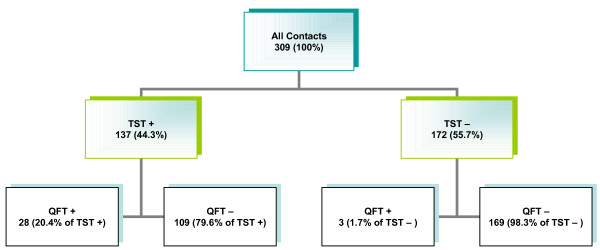
Application of the QFT-G test in a population of close contacts

Overall, 137 of the 309 contacts (44.3%) developed an induration greater than 5 mm at the TST site and of these 28/137 (20.4%) were positive by QFT-G, whereas 3 of the 172 (1.7%) who had a negative TST result were QFT- G positive (Figure 1). Employing other TST cut offs, 64/309 (20.7%) had an induration of more than 10 mm, and 25 (8.1%) of more than 15 mm. According to the current German guidelines [20], the lowest of these three sizes was taken as a cut off for positivity in the test. The patients with positive TST results had a mean age (± SD) of 29.0 (± 11.1) years which did not differ from those with negative TST (28.1 ± 10.0, n.s.); they were also similarly distributed by sex, but they differed in respect of origin: 58/84 (69.0%) foreign-born persons gave a positive TST result, compared with 79/225 (35.1%, *p*<0.001) of those born in Germany.

**Figure 2:**
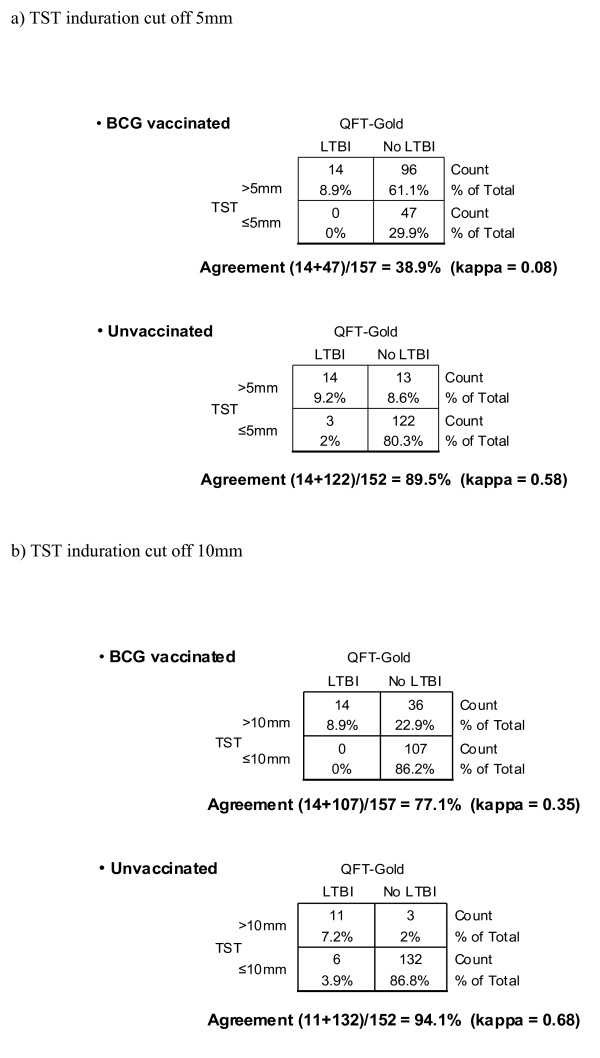
Effect of BCG vaccination on agreement of TST and QFT-G as shown in 2x2 contingency tables : a) TST induration cut off 5 mm b) TST induration cut off 10 mm

Of all contacts, 157 of 309 (50.8%) had received BCG vaccination (see Figure 2). Of these, 110 (70.1%) gave a positive TST result. However, of the 152 contacts who had not received BCG vaccination, only 27 (17.8%) gave a positive TST result (*p*<0.0001). Fifty of the 84 (59.5%) foreign-born had been previously BCG-vaccinated, compared with 107/225 (47.6%) of those born in Germany (n.s.). For all positive induration sizes (i.e. >5 mm), the mean induration size (± SD) of the TST was 11.2 (± 4.3) mm in the BCG-vaccinated TST-positives and 12.1 (± 4.9) mm in the unvaccinated contacts, but this difference was not statistically significant. Thirteen contacts (4.2%) had an induration size of 5 mm and just failed to reach a positive result (= 6 mm), and 16 (5.2 %) had a response of exactly 6 mm. However, there were only two QFT-G results (0.38, 0.39, both TST positive at 10 and 11 mm) close to its cut off of 0.35 IU/ml: Most of the negative results were 0.0 (n = 138) or nearly 0, the mean of positive results was 7.37 IU/ml (ranging from 0.38–21.43 IU/ml).

In contrast to the high rate of TST positivity as described above, only 31 of the 309 contacts (10%) showed a positive QFT-G result. For those with a history of BCG vaccination, none of the 47 TST-negative contacts were QFT-G positive, and 14 of 110 TST-positive contacts (12.7%, *p *= 0.01) were positive in both tests (see Figure 2 a). For those contacts not BCG vaccinated, 14 of 27 TST-positive contacts (51.9%) and 3 of 125 non-vaccinated TST-negative contacts (2.4%, *p*< 0.0001) were QFT-G-positive.

Overall agreement between TST and QFT-G was low (κ = 0.20, 95% CI 0.14–0.23;), with concordant results in only 197/309 (63.8%). Broken down by TST result, this corresponded to concordance in 169/172 (98.3%) contacts with TST-negative results, but only 28/137 contacts (20.4%) with TST-positive results. Concordance between the two tests was poor in those BCG vaccinated (38.9%; κ = 0.08, 95% CI 0.026–0.08), but was high (89.5%; κ = 0.58, 95% CI 0.4–0.68) in those who had not been vaccinated (Figure 2 a). There was no correlation between either TST-positive or QFT-G-positive results and the different times of exposure of the contacts to their source cases (data not shown).

We also examined the possibility that adopting a higher cut off point for positivity in the TST might also result in higher levels of agreement between the two tests. Following the current guidelines of the American Thoracic Society [21], we raised the cut off of a positive result from 5 to 10 mm, a cut off diameter that is normally recommended for persons who are at increased risk, but are not close contacts. This caused an increase in the κ value between TST and QFT-G response in the BCG-vaccinated contacts to0.35 (95% CI 0.24–0.35) and, for the non-vaccinated contacts, to 0.68 (95% CI 0.46–0.81) representing strong agreement. However, even when applying a 10 mm cut off for the TST (Figure 2 b), more of the non-vaccinated contacts were QFT-G-positive (17/152; 11.2%) than positive by the TST (14/152; 9.2%, n.s.), suggesting at least a trend to a greater sensitivity for the QFT-G test. In contrast, for those BCG-vaccinated, a similar rate of QFT-G-positive responses were seen (14/157; 8.9%), but significantly more (p < 0.001) were positive by the TST (50/157; 31.8%), suggestive of poor specificity of the TST in the BCG-vaccinated group.

In the logistical regression analysis, the estimated odds of having a positive TST result in close contacts (at a cut off of 5 mm) were nearly 13 times higher (OR 12.6, 95% CI 6.9–22.7; *p *< 0.0001) in BCG-vaccinated contacts (Table 2 a), while foreign origin led to an fivefold increase in risk (OR 5.4, 95% CI 2.7–10.6, *p *< 0.0001). At a cut off of 10 mm (Table 2 b) the odds of having a positive TST result in BCG-vaccinated was reduced but still highly significant (OR 4.8, 95% CI 2.3–9.6, *p *< 0.0001). The QFT-G test results, however, were only associated with foreign origin as an independent predictor (Table 3).

**Table 2:** Results of multiple logistical regression: Odds ratio for a positive TST (tuberculin skin test) result

**a)**			
TST (5 mm cut off)	Odds ratio	95% confidence interval	*p*

BCG vaccination	12.6	6.9–22.7	<0.0001
Age	1.0	0.98–1.04	0.5 (n.s.)
Sex	1.3	0.72–2.24	0.4 (n.s.)
Origin (Foreign/ German)	5.4	2.7–10.6	<0.0001
Previous contact tracing	1.7	0.3–10.9	0.6 (n.s.)
Exposure time	1.0	0.99–1.0	0.6 (n.s.)

**b)**			

TST (10 mm cut off)	Odds ratio	95% confidence interval	*p*

BCG vaccination	4.8	2.3–9.6	<0.0001
Age	1.05	1.01–1.08	0.005
Sex	1.5	0.8–2.9	0.2 (n.s.)
Origin (Foreign/German)	7.3	3.7–14.3	<0.0001
Previous contact tracing	0.6	0.05–7.4	0.7 (n.s.)
Exposure time	1.0	0.99–1.0	0.99 (n.s.)

**Table 3:** Results of multiple logistical regression: Odds ratio for a positive QFT-G (QFT-Gold) result

QFT-G	Odds ratio	95% confidence interval	*p*
BCG vaccination	0.7	0.3–1.4	0.29 (n.s.)
Age	1.0	0.98–1.1	0.5 (n.s.)
Sex	1.4	0.6–3.2	0.4 (n.s.)
Origin (Foreign/German)	4.7	2.1–10.5	<0.0001
Previous contact tracing	3.9	0.6–24.1	0.1 (n.s.)
Exposure time	1.0	0.99–1.0	0.8 (n.s.)

All individuals with a positive QFT-G and a positive TST were offered nine months of INH treatment for LTBI with a daily uptake of 300 mg INH as recommended by the current German guideline [20]. Of these 28 persons, 15 (54%) accepted this offer. None of the contacts persons has developed a TB disease up to now.

## Discussion

In a routine contact investigation setting, our findings showed an increase in the incidence of TST positive reactions in BCG vaccinated persons, while QFT-G was unaffected. Both tests had similar rates of positive result in unvaccinated persons, and were more frequently positive in foreign born individuals predominantly from high TB incidence countries.

While some recently-published studies showed that the degree of exposure of contacts to the source case is usually more closely correlated with a positive result in a whole blood IFN-γ assay [8,10-13] or the detection of MTB-specific T-cells in an ELISPOT [22-24] than with a positive TST reaction, we investigated possible differences between TST and QFT-G test results within the same risk level of exclusively close contacts. Neither test showed clear correlation with estimated time of exposure. However, all contacts had a minimum of 40 hours of exposure to their respective index case and were thus deemed "close contacts". A possible explanation for this finding is that there is only a small increase in the chance of becoming infected during exposure times exceeding 40 hours.

Since the TST is still regarded by many as the gold standard for the diagnosis of LTBI, official guidelines [20,21] currently require that a positive test result in close contacts should be followed by treatment. However, the TST only offers an indirect diagnosis of LTBI and can lead to a substantial number of false-positive test results, decreasing its PPV [13]. This contact study of infectious TB source cases in a German metropolis reveals two crucial points that affect a decision to start treatment for presumed LTBI on the basis of a positive TST. First, in absolute numbers, nearly one half (137/309, 44.3%) of the close contacts were TST-positive, but there were more than four times as many TST-positive persons among those contacts who had previously been BCG-vaccinated (110/137 = 80.3% vs. 27/137 = 19.7%, *p *< 0.0001) indicating the likelihood of a false positive reaction due to cross-reactivity with *M. bovis *BCG strains. Secondly, we found that more than one-half of the contacts (157/309, 50.3%) had previously been BCG vaccinated, and this was independent of their origin (German or foreign-born) and their age, because even in Germany BCG vaccination was recommended up to March 1998.

Of note was the finding that persons who were BCG vaccinated and QFT-G-positive were more likely to have TST responses >10 mm than those QFT-G-positive, but unvaccinated. This suggests that BCG vaccination plays an important role in the routine immunological memory of MTB infection, and may prime the immune system to respond more strongly to the TST after MTB infection. In this situation accepting the QFT-G response as the gold standard validates a 10 mm TST cut off for BCG vaccinated persons as the minimum induration size on which a decision for treatment for LTBI can be based.

As one might expect, our study demonstrates – with a κ value of 0.08 – a poor correlation between the results of TST and those of the QFT-G assay among BCG-vaccinated contacts. However, it was surprising that even in unvaccinated contacts QFT-G failed to confirm nearly 50% of positive TST results – there were only 14 QFT-G-positives (52%) among 27 TST-positive contacts – when the induration cut off for the TST was set to 5 mm diameter. This indicates at least four possible explanations.

The first is that QFT-G is not sensitive enough to confirm true LTBI, thus producing false negative IFN-γ -assay results. However, there is no evidence for this assumption, because in three contacts there were positive QFT-G results while their TST response was negative, whereas the rest of the TST/QFT-G-negative group showed excellent concordance (122/125, 97.6%). The second possibility is that the 5 mm cut off of TST representing a positive result, used to achieve increased sensitivity in high-risk persons, is too low, because of an overestimation of the true intensity of exposure of a contact and must therefore be raised. The fact that increasing the cut off to 10 mm resulted in a strong agreement (κ = 0.68) between the results from TST and those from QFT-G in the non-vaccinated population seems to support the influence of BCG as a confounder of the TST. There were only 3 (2%) QFT-G negative but TST positive responses >10 mm in the non-vaccinated group, and 6 (4%) QFT-G positive but TST negative, indicating QFT-G is equally or more sensitive than the TST in non-vaccinated persons. This corresponds to the results of other studies, in which a strong agreement between TST and ELISPOT could be seen when a diameter of 10 mm was used as cut off from the start [25]. Only 3 of the 13 non-vaccinated contacts with TST responses between 5 and 10 mm were QFT-G positive, suggesting that QFT-G was able identify those with TST responses in this range that were truly infected with MTB.

The third possibility is that, as in the case of BCG vaccination, there are cross-reactivities with other mycobacteria. Antigen preparations from non-tuberculous mycobacteria (NTM; generally *M. avium *or *M. intracellulare*) have been used in a large number of studies and countries to determine the rate of immune reactivity to NTM in various populations [26-30]. These studies indicate that asymptomatic NTM infections are common, and can be responsible for up to or more than 50% of 5–14 mm and as high as 19% of = 15 mm PPD reactions in low TB incidence populations. Thus, following the published studies worldwide on this topic, TST positivity of 8.6% of the close contacts who are QFT-G-negative and not previously BCG vaccinated in our study, could explain a number of false positive reactions, as due to NTM.

The fourth possibility is that the QFT-G test, which relies on the presence of antigen-dependent immediate effector T-cells, is detecting current or more active infection, while the TST, which can detect central memory T cell responses is detecting past, dormant or resolved infection [31].

Of course, some contacts are definitely known *a priori *not to have been BCG-vaccinated and to have been very intensively exposed, e.g. by intimate contact with a source case within the family. If there is such evidence, a positive TST result will generally be sufficient to determine MTB infection. Such information, however, is rarely provided in the often complex mix of different settings and contact numbers in routine contact tracing. This is especially the case as BCG is normally given at infancy and therefore most people cannot recall having received it, and searching for a BCG scar might be an insensitive indicator of vaccination. Therefore, MTB-specific whole-blood INF-γ tests should be recommended as a confirmation for a given TST result, if a subsequent INH treatment for LTBI is to be taken into consideration. Increasing the cut off for a positive TST to 10 mm will further increase the probability of a true MTB-infection, but even in this case some true MTB infections will be missed, as was probably the case in 2.4% (3 QFT-G-positive, but TST-negative) of the 125 TST-negative close contacts in our study population.

While sensitivity cannot be formally tested in LTBI, the comparison of QFT-G with TST in BCG unvaccinated contacts indicates similar sensitivity for LTBI. Limited studies [12] have shown QFT-G-positive individuals do develop active tuberculosis, but the PPV for a QFT-G test is not yet established. Clearly, true LTBI is a prerequisite for subsequent TB disease, and if disease cases derive from a smaller number of positive QFT-G subjects the PPV will be higher than for the TST. But, while there are reasons for contacts to be QFT-G- positive and TST-negative which are evident in the present study, the clinical outcome for such persons in the absence of treatment for LTBI will determine the final value of the QFT-G test.

In conclusion, the data presented here suggest that a MTB-specific whole-blood INF-γ test (QFT-G) appears to be more valid method for screening recent contacts for LTBI, especially when a large number of contacts have previously been BCG-vaccinated or their BCG vaccination status cannot be accurately determined. QFT-G also has benefits over the TST if contacts have migrated from foreign countries, where NTM infections are prevalent. Owing to the high specificity of this IFN-γ test, the ESAT6/CFP-10/TB7.7 based QFT-G assay allows better discrimination between true infection and cross-reactivity, and can thus circumvent the unpredictable influence of BCG and NTM on the TST. Availability of MTB-specific whole-blood INF-γ tests – more accurate than the TST – could lead to a better chance of true positive test results and thus, in turn, to more systematic use of treatment for LTBI. In addition, it is suggested that if the TST is to be used, the cut off should be raised from 5 mm to 10 mm, even for close contacts, in order to minimize the large number of false positive results seen at this cut off.

## Competing interests

The author(s) declare that they have no competing interests.

## Authors' contributions

RD and TS designed the study and wrote the manuscript. RD and AN carried out and interpreted the statistical analysis. CL and MF participated in the design of the study and the data interpretation. KM recruited patients, obtained medical data and assessed results. All authors read and approved the final manuscript.

**Figure 3:**
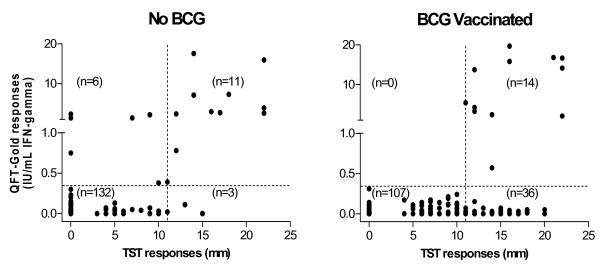
QFT-G and TST responses for contacts, stratified by BCG vaccination status. Vertical dotted lines represent a > 10 mm cut of for the TST, and horizontal dotted lines represent the QFT-G cut off.
